# Road traffic incidents in Uganda: a systematic review of a five-year trend

**DOI:** 10.5249/jivr.v9i1.796

**Published:** 2017-01

**Authors:** Joseph Kimuli Balikuddembe, Ali Ardalan, Davoud Khorasani-Zavareh, Amir Nejati, Kasiima Stephen Munanura

**Affiliations:** ^*a*^Department of Disaster Public Health, School of Public Health, Tehran University of Medical Sciences, Tehran, Iran.; ^*b*^Tehran University of Medical Sciences – International Campus, Tehran, Iran; ^*c*^National Institute of Health Research, Tehran University of Medical Sciences, Tehran, Iran.; ^*d*^Harvard Humanitarian Initiative, Harvard University, Cambridge, USA.; ^*e*^Safety Promotion and Injury Prevention Research Center, Shahid Beheshti University of Medical Sciences, Tehran- Iran.; ^*f*^Department of Health in Disaster and Emergency, School of Health Safety and Environment, Shahid Beheshti University of Medical Sciences, Tehran, Iran.; ^*g*^Department of Clinical Science and Education, Karolinska Institute, Stockholm, Sweden.; ^*h*^Emergency Medicine Research Center, Department of Emergency Medicine, Imam Khomeini Hospital, Tehran University of Medical Sciences, Tehran, Iran.; ^*i*^Directorate of Road Traffic and Road Safety, Uganda Police Force, Kampala – Uganda.

**Keywords:** Systematic review, Traffic crashes, Injury, Trauma, Uganda

## Abstract

**Background:**

Over the years, Uganda has been one of the low and middle-income countries bearing the heaviest burden of road traffic incidents (RTI). Since the proclamation of the United Nations Decade of Action for Road Safety 2011 – 2020, a number of measures have been taken to reduce the burden. However, they ought to be premised on existing evidence-based research; therefore, the present review ventures to report the most recent five-year trend of RTI in Uganda.

**Methods:**

Based on Preferred Reporting Items for Systematic Reviews and Meta-Data Analysis (PRISMA) guidelines, a systematic review was employed. Using a thematic analysis, the articles were grouped into: trauma etiology, trauma care, mortality, cost, trauma registry and communication, intervention and treatment for final analysis.

**Results:**

Of the nineteen articles that were identified to be relevant to the study, the etiology of RTI was inevitably observed to be an important cause of injuries in Uganda. The risk factors cut across: the crash type, injury physiology, cause, victims, setting, age, economic status, and gender. All studies that were reviewed have advanced varying recommendations aimed at responding to the trend of RTIs in Uganda, of which some are in tandem with the five pillars of the United Nations Decade of Action for Road Safety 2011 – 2020.

**Conclusions:**

Peripheral measures of the burden of RTIs in Uganda were undertaken within a five-year timeframe (2011-2015) of implementing the United Nations Decade of Action for Road Safety. The measures however, ought to be scaled-up on robust evidence based research available from all the concerned stakeholders beyond Kampala or central region to other parts of Uganda.

## Introduction

The low and middle-income countries (LMICs) bear the heaviest burden of all road traffic incidents (RTIs) in the world.^1^ This is noted at 85% of the global average of 750,000 compared to 15% in high-income countries. These RTIs have caused approximately 1.3 million deaths, and 20 to 50 million injuries. ^2,3^ RTIs at times are interchangeably recorded as road traffic injuries, road traffic crashes, road traffic accidents, or motor vehicle crashes. However, each may slightly differ from the others. The present review defines road traffic injuries according to the World Health Organization (WHO) as the cause of fatalities leading to loss of life during the event time or a maximum of 30 days after the crash. ^4^ The African region (AFRO) alongside the Eastern Mediterranean region (EMRO) have the highest rate of road traffic deaths, which are described as a global pandemic and development problem.^5^

Over years, Uganda has not been spared from the heavy burden of RTIs. At present, it experiences RTI deaths at 28.9 per 100,000 population. This is quite concerning as it even exceeds the 24.1 per 100,000 population for the AFRO and 18.0 per 100,000 population global average for deaths respectively. As a result, Uganda is among the top-ranking countries for RTIs along with South Africa, Nigeria, Iran, Thailand and Dominican Republic at 31.9; 33.7; 34.1; 38.1; and 41.7 per 100,000 population, respectively.^6^

The trend of Uganda’s RTIs is further mirrored by the solid evidence denoting traffic injuries within the top-ten causes of mortality in the country.^7^ Accordingly, not less than 1,000 and 10,000 victims were killed and injured respectively due to RTIs in Uganda between 2010 and 2013. This has been reported in the annual traffic and crime reports of the Uganda Police Force (UPF). ^8-11^ This has cost Uganda dearly particularly in terms of the loss of a significant proportion of its economically active population, which in turn retards its economic growth and development. In response, a number of measures to reduce the burden of RTIs have been initiated ever since the United Nations Decade of Action for Road Safety 2011 – 2020 was proclaimed on 11th May 2011. ^1,6^ However, there’s still a great need for establishing the existing RTI trend in Uganda based on the evidence-based research in order to properly inform any measures undertaken to reduce them. For this specific reason, the present study has been undertaken to address this need to some extent.

This review reports on the five year trend of RTIs in Uganda from 2011 and 2015. The results of the study provide useful information for all concerned stakeholders to enable them to better respond to RTIs. This will be vital for the remaining five years of implementing the United Nations Decade of Action for Road Safety.

## Methods

**Systematic Review **

Original articles published electronically from 2011 to the present were reviewed using the Preferred Reporting Items for Systematic Reviews and Meta-Data Analysis (PRISMA) guidelines.^12^ A period of 2011 – 2015 was chosen to critically study the trend of RTIs in Uganda as it covers the middle point of implementing the United Nations Decade of Action for Road Safety which binds all countries to halve their traffic crashes. The entire review process that led to this paper was conducted between November 2014 and April 2015.

**Search strategy**

We first searched in both electronic databases and journal collections. They included: Google scholar, Medline, PubMed, Science Direct, OVID, John Wiley, Web Science and Springer by using the text words such as (“road traffic injury” OR “road traffic” OR “accident”) AND (“mortality” OR “death” OR “trauma”) AND (“Uganda” OR “Kampala”). We also manually searched the “Google” search engine by interchanging the above text words with each other. Finally, we used the reference citations in some articles for a further search.

**Data extraction**

At first, all article references (title, abstract and keywords) that we deemed relevant to the topic were read, retrieved and stored using EndNote software X7. Their details were sub-grouped into title, year, setting, variable/division, design, database and findings. Afterwards, the full text of each article was electronically downloaded.

**Inclusion and exclusion criteria**

Selection for each study depended on a predefined inclusion and exclusion criteria. The inclusion criteria took into consideration the following: articles focusing on RTIs or road safety, a study denoting Uganda (directly and indirectly), original articles with a study design published in English, and articles published in 2011 and onwards. The exclusion criteria excluded the following: studies not focusing on either road traffic incidents or road safety, topics not focusing on Uganda, articles without or an inadequate study design, articles published before 2011 and articles not published in English. Case reports and case series, conference proceedings, letters and commentaries were also excluded.

**Quality appraisal**

 The study was typically a systematic review done for purely philanthropic purposes. It therefore did not require any sanctioning or approval from any authority. However, to ensure the validity, at first, reference extractions were independently checked and screened by two authors in order to identify and prevent any duplication. This is because some articles were reportedly published in more than one journal database. Again, two authors reviewed the references to decide whether they met the inclusion criteria. At a certain point, however, some discrepancies regarding the eligibility of some of the articles emerged, especially with the “not-related articles”. In this case, more than two authors resolved the discrepancies in order to ensure the consistency.

## Results

**Systematic literature review**

**Figure 1:**
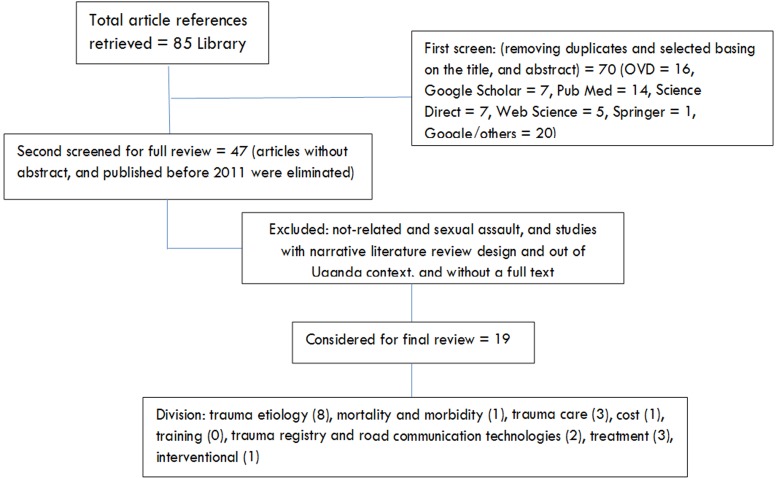
A PRIMSA flow chart indicating steps followed in retrieving and screening the articles for review

The initial search yielded a total of 85 articles. Three screenings and reviews were conducted as indicated in the PRISMA flow chart below in Figure 1. First, we eliminated all the title duplicates using EndNote software command “Find Duplicates” and then the “eliminate duplicate” command, while delineating the titles and abstracts which were potentially related to the topic. This led to 70 studies. Second, the articles with only abstracts and published before 2011 were excluded and this reduced the total to 47 articles. At this point, a full text of the articles were obtained for a detailed review. Afterwards, with an aid of the Microsoft Excel spreadsheet, the articles were sub-categorized into 10 divisions: not-related, sexual assault, interventional, training, cost, trauma care, trauma etiology, mortality and morbidity, trauma registry and road communication technologies, and treatment. This aimed at facilitating the analysis process.

It should however be noted that, articles qualified for the final review were sub-grouped in only seven divisions (Table 1). The second screen involved foregoing articles categorized as “not-related” and “sexual assault”, and those that were narrative and literature review in nature. Also, the articles out of Ugandan context, and one whose full-text couldn’t be retrieved were foregone. In the end, 19 articles were eligible for the final review, and the details were used (Table 1).

**Table 1:** Details of articles that were finally included for review

Author and year	Title	Setting	Division	Design /instrument	Database/ journal	Finding
Otieno et al (2011)	Pre-hospital and First Health Facility Management of Patients with Severe Musculoskeletal Injuries in Uganda	Mulago Hospital Accident and Emergency Unit (MHAEU)	Trauma care	Cross-sectional descriptive analysis (structure questionnaire)	Google Scholar	Minimal care is administered in the field to musculoskeletal injury patients due to inadequate resources and equipment and along with unskilled responders.
Kigera et al (2011)	Injury to presentation delays among musculoskeletal trauma patients in Uganda	Mulago Hospital MHAEU	Trauma care	Prospective study (interview and case notes)	Google Scholar	The median delay in presentation was 3.5 hours associated with factors of patients being managed as an outpatient, having an upper limb injury, being less than 18 years of age and involved in RTCs.
Kitara D. L (2011)	Boda Boda Injuries in Gulu Regional Hospital, Northern Uganda	Gulu regional hospital, Northern Uganda	Trauma etiology	Retrospective study (medical records)	Google Scholar	Boda Boda injuries constituted (21.9%) of all admissions due to trauma and majority (57.5%) of the victims were males
Muto et al (2011)	Unintentional childhood injury patterns, odds, and outcomes in Kampala City: an analysis of surveillance data from the National Pediatric Emergency Unit	National Pediatric Emergency unit in Kampala city	Trauma etiology	A cross sectional analysis (data registry)	OVID	Unintentional injuries are the common causes of hospital visits by children <13 years (especially boys). Homes, roads and educational facilities are commonest unintentional injury sites.
Hsia et al (2011)	Epidemiology of child injuries in Uganda: Challenges for health policy	Mulago hospital	Trauma etiology	Records of trauma registry	Google Scholar	The most common causes of injuries were traffic crashes (34%), falls (18%) and violence (15%).
Jayaraman et al (2011)	Disparities in Injury Mortality Between Uganda and the U.S: Comparative Analysis of a Neglected Disease	Uganda (Kampala), the U.S and San Francisco	Mortality	Database of deaths	OVID	Urban injury-related mortality is significantly higher in Uganda than in the United States.
Maryse et al (2012)	Corruption in the health care sector: A barrier to access of orthopaedic care and medical devices in Uganda	Uganda as a whole	Trauma care	Qualitative case study (open -ended interviews)	Web science	Poor leadership in government and corruption were the major barriers to access orthopaedic care and orthopaedic medical devices.
Trond et al (2012)	Cultural and socio-demographic predictors of car accident involvement in Norway, Ghana, Tanzania and Uganda	Norway, Ghana, Uganda and Tanzania	Trauma etiology	Questionnaires	Science Direct	Male gender was the only significant predictor of accident involvement in Norway. Introverted and extroverted culture, and destiny orientation as well as male gender written culture were associated with accident involvement in the African countries.
Derek et al (2012)	Road Communication Technologies and Safety Regulation Enforcement on Roads in Uganda	Uganda- countrywide	Trauma Registry	Questionnaires	Google Scholar	RCTs can play a significant role in enforcement of road safety.
Mujuni et al (2012)	Acute traumatic coagulopathy among major trauma patients in urban tertiary hospital in sub Saharan Africa	Mulago Hospital MHAEU	Treatment	Prospective cohort (Laboratory based)	Web science	The commonest cause of injury was RTC, followed by assaults, burn and falls. ATC was strongly associated with acute renal injury.
Douglas et al (2013)	Using baseline and formative evaluation data to inform the Uganda Helmet Vaccine Initiative	Kampala	Interventions	Qualitative methods (Focus group discussions, interviews and observation)	Pub Med	During motorcycle crash, many boda boda riders in Kampala continue to ride without elements and barriers were noted as uncomfortable, ‘Helmet is too hot’, ‘Helmet is too expensive’, and ‘Helmet is of low quality’.
Trond et al (2013)	Culture related to road traffic safety: A comparison of eight countries using two conceptualizations of culture	Norway, Russia, Ghana, India, Uganda, Tanzania, Turkey and Iran	Trauma etiology	Self-mailed questionnaire	Science Direct	Norwegians reported overall safer attitudes towards traffic safety and driver behavior than remaining country clusters. Individuals in Africa reported the highest risk perception. The countries also differed substantially in road traffic culture as symbol use.
O’Hara et al (2014)	The Orthopaedic Trauma Patient Experience: A Qualitative Case Study of Orthopaedic Trauma Patients in Uganda	Mulago National Referral Hospital in Kampala	Cost	Descriptive qualitative case study (structure interviews)	Pub Med	Limited resources to fund the treatment of orthopaedic trauma patients in Uganda leads to reliance of patients on their friends, family, and hospital connections, and a tremendous economic burden that falls on the patient and their dependents.
Munabi et al (2014)	Musculoskeletal disorder risk factors among nursing professionals in low resource settings: a cross-sectional study in Uganda	Five hospitals through Uganda	Trauma etiology	Cross- sectional study (questionnaire)	Pub Med	Significant risk factors for MSD include: being absent from the work station for more than 6 months due to illness or an accident, working in awkward postures, pushing /pulling of heavy loads and mental exhaustion.
Musiitwa et al (2014)	Emergency Ultrasound Predicting the Need for Therapeutic Laparotomy among Blunt Abdominal Trauma Patients in a Sub-Saharan African Hospital	Mulago Hospital MHAEU	Trauma etiology	A prospective observational study (pretested questionnaire)	Pub Med	The commonest injuries were to the head 80 (42%) and the abdomen 54 (28%). A SSORTT score of >2 appropriately identified patients needed a therapeutic Laparotomy.
Twinomuhangi et al (2014)	Application of geographic information systems methodology to injury surveillance in Uganda	Two referral hospitals (Mulago and Mubende) in Central Uganda	Trauma Registry and communication	Trauma registry form	Google Scholar	GIS-based surveillance and mapping capacity may become an important approach to treating the prevalence of different types of injury in Uganda over the longer term.
Okello et al (2014)	Serum lactate as a predictor of early outcomes among trauma patients in Uganda	Emergency Department of Mulago Hospital	Treatment	Cross-sectional analytical study (medical observation and theater operation)	Pub Med	Serum lactate ≥2.0 mmol/l had a hazard ratio of 1.10 (p <0.001) for ED disposition, 4.33 (p = 0.06) for the 72-h non-discharge disposition, and 1.19 (p <0.0 01) for 72-h mortality.
Daniel et al (2014)	Upper extremity injury management by non-physician emergency practitioners in rural Uganda: A pilot study	Karoli Lwanga Hospital Emergency Centre Rukungiri District	Treatment	Retrospective querying of EC quality assurance database	Science Direct	Non-physician clinicians appear to be performing upper extremity repairs with good outcome and key variable for success was initial injury type.
Michael et al (2015)	Distribution and characteristics of severe traumatic brain injury at Mulago National Referral Hospital in Uganda	Mulago National Referral Hospital	Trauma etiology	Retrospective chart and medical record review	Science Direct	Motorcycle road traffic accident was the leading mechanism of injury and male ages 15 - 29 were predominant demographics.

**Literature review designs**

Qualitative approaches which included: observations, questionnaires, interviews, laboratory and theater operation reports, medical notes, records, registries, charts and databases were the main sources reported in the reviewed articles. We noticed that the majority of the studies were published in 2011 and 2014 with a focus on trauma etiology, trauma care, mortality, cost, trauma registry and communication and treatment as shown in Table 1. Six studies apportioned traffic crash trauma etiology as an important cause of injuries in Uganda based on the type of crash, injury physiology, cause, victims and setting as well as the age, economic status, and gender of the victims. Some studies highlighted other causes of injury in Uganda for instance: assault, gunshot, burns, falls, animal and snake bites, sports, poisoning, stabs and cuts. 

**Risk factors of RTIs**

Some underlying risk factors of RTIs were reported in five studies. They included: extroverted and introverted road use culture; reckless driving; inappropriate driving experience; lack of respect to road traffic laws and vehicle roadworthiness. Others are road infrastructure deficiencies and inappropriate usage, alcohol and drug impairment, not wearing appropriate safety gear (helmet, seat belts and child restraints), speeding and overloading vehicles.

**Injuries associated with RTIs**

Injuries of varying degrees of severity (mild, serious and life threatening) were reported in 15 articles. They included: upper-extreme- musculoskeletal and spine injuries for instance; head, neck, chest and abdominal injuries as well as fractures (femur and tibia). Moreover, 16 studies reported RTIs as a direct cause of injury in Uganda whilst 12 studies underscored RTIs injuries and trauma as top-ranking causes of death in Uganda.

**Distribution of RTIs by gender and age**

Eight studies directly reported that the males were at far greater risk of being involved in a RTI than the females. All road users: passengers, pedestrians, motorists and cyclists were noted to be prone to RTIs in 10 studies. However, children aged 0 -15 years were cited in 8 studies as being highly vulnerable to RTIs especially in the urban setting. Boda-bodas (commercial motorcycles) were cited as a significant cause of RTIs in Uganda in 7 studies.

**Scope of RTIs**

Furthermore, Kampala, the capital city, dominated the study scopes. In this regard, so far 13 studies have been conducted mainly at Mulago National Referral hospital, which is close to Kampala. However, 2 studies were conducted at Gulu and Rukungiri in Northern and Western Uganda respectively. More, three studies compared RTIs in Uganda with other countries (United States of America, Norway, India, Iran, Ghana, Tanzania and Turkey).

**Advanced recommendations**

Fortunately, we reported that all of the studies advanced some recommendations pertaining to both road safety and trauma care for road traffic victims. They included: prompting context-specific policies focused on cultural practices and beliefs and establishing and improving access to formal pre-hospital trauma care services. Additionally, other recommendations included proper training and licensing of motorists and cyclists, surveillance and enforcement of road traffic and safety laws, upskilling and training of lay responders, promoting the use of helmets among motorcyclists and increasing funding and human resources where suggested. Others agitated for the strengthening of clinical capacities, addressing the rising burden of high trauma care cost (especially for the poor), and integrating different road communication technologies in road safety promotion along with further research.

Two studies were related to the use of registries and road communication technologies in the quest for promoting the road safety. In addition, it should also be noted that one study on the trauma care cost, one on policy intervention on trauma care, and three studies on trauma care treatment were considered for a review since they divulged some useful facts on the subject matter as indicated in Table 1 findings.

## Discussion

In the first place, our findings show that the causes of RTIs in Uganda are many. Therefore, any stern measures intended to counteract them should also be multi-dimensional in nature. The majority of the studies considered for this paper were published in the immediate aftermath of the Decade of Action proclamation (after 2011). To some extent, this shows that there has been a serious attempt to understand and reduce the carnage caused by RTIs. As one of the biggest public health burdens affecting Uganda and the whole world, it needed to be championed. Indeed, this was demonstrated in the zealous research undertaken in the arena of trauma etiology, care, mortality and morbidity, intervention, treatment and registries. However, the majority of the studies were hospital based. There is still a great need to carry-out other RTI field related research across Uganda.

Although it’s not conclusive and may warrant more in-depth research, we have noted that to a certain degree some research efforts considerably contributed to a decline in the number of RTIs in Uganda. This is evidenced by the annual crime and road traffic reports of UPF indicating a decline in RTIS. For example 22,272 cases were reported in 2011, 19,870 in 2012 and 18,368 cases in 2013.^8-11^ However, given that we are now at the mid-point of the Decade of Action for Road Safety, more research needs to be encouraged and recommendations followed up on.

So far, the heightened risk factor of RTIs in Uganda reported in various studies is also consistent with risk factors reported annually in a wide body of literature like the official reports of UPF and WHO. Some of these include: inappropriate road use culture, reckless driving, lack of respect for road traffic laws, inconsiderate use of roads, alcohol and drug impairment, and speeding. These factors are often exacerbated by the lack of a systematic approach in RTI prevention,^13^ which is common in many low and middle-income countries.^14-16^ To address this matter, national and worldwide concerted efforts involving all concerned stakeholders are needed. In this regard, so far the road safety measures undertaken by the Ugandan government and UPF in unison with the WHO, academia, civil society and other players need to be applauded. However, in the interests of achieving much more progress towards the road safety, some challenges still require much more attention as they remain obstacles to the effective road safety in Uganda. Among those noted in reviewed articles were culture, corruption, funding constraints, inadequate awareness and infrastructure,^17-19^ which is again consistent with other findings in low and middle-income countries.^14,15,20^

The results clearly demonstrate that RTIs are the leading cause of death in Uganda. They also show that of different types of injuries, head, chest and abdominal injuries are the most life-threatening injuries to the victims as noted in some studies.^21,22^ RTIs are ranked among the top-ten causes of mortality for all ages in Uganda alongside malaria, respiratory infection, anemia, meningitis, tuberculosis and HIV/AIDS to mention but a few.^7^ In respect of the very high death toll from RTIs, it should be noted that pre-hospital setting is by far the most predominant place where 85% of deaths occur in many LMICs^23,24^ and RTIs in Uganda are no exception. However, it’s irrefutable that timely pre-hospital care can reduce injury severity reducing the trend of RTIs deaths by saving lives, treating injuries efficiently and effectively, preventing infections and injury-related diseases as well as preventing disabilities.^25,26^ This is why some research has called for the establishment of structured and organized trauma care systems as one way of averting avoidable deaths in Kampala and elsewhere.^20,27-29^

Furthermore, given that each group of road users is exposed to different risk factors from RTIs, a trimodal approach should be employed in guiding road safety in Uganda. In this case, all ages and genders (male and female); settings (urban and rural) and economic status-quo should be considered, especially when it comes to low or middle income areas.^16^ For instance, children 15 years of age and younger are cited to be particularly exposed to RTIs at Kawempe, Kampala's suburbs. ^30^ Therefore, promoting road safety sensitization and education is not only needed in schools but it’s also needed in homes vis-à-vis at a family level. Where possible, parents should provide necessary accompaniment and supervision for their children while on the road.

In the same vein, the high RTI risk posed by boda boda is inevitable given Uganda’s underdeveloped transport system. We note that, boda boda transport mode is fairly affordable for the majority of Ugandans compared to more expensive motorized transportation. Indeed, this is in line with a study that observed how cheap motorcycle transport in Nigeria is a serious contributory factor to RTIs.^31^ Despite its affordability and being a quick means of transportation, boda-bodas have emerged as a silent killer of many Ugandans despite the efforts made to mitigate the problem.^32^

Ideally, effective information management of RTIs enhances timely data retrieval and control actions. Accordingly, data paucity and the lack of official RTI data registries have been consistently recognized as one of the major challenges preventing the road safety improvement in Uganda.^26^ In light of this, the two trauma registry and communication studies depict an exponential need for encouraging the establishment of more RTI dedicated registries and databases to facilitate timely retrieval of updated information and statistics. This notwithstanding is pleasing to note that at least the role of RTC for example Geographic Information Systems (GIS) is being embraced as an effective tool in the promotion of road safety in Uganda. No doubt, information technology offers some enormous opportunities in the quest of ushering effective road safety and injury surveillance in Uganda.

By and large, the scope of many studies revolved around Kampala, the capital city of Uganda, in the central region. On one spectrum, we argue that this can be attributed to an upsurge of rapid motorization taking place in Kampala to some extent, as well as the ready availability of information and resources needed for research which can be obtained in Kampala. However, we have also noted with particular concern that, albeit the majority of RTIs being annually registered in Kampala in particular and the central region in general, other parts of Uganda are equally affected by RTIs.^27^ With this in mind, facilitating countrywide research on RTIs beyond Kampala and the central region to northern, western and eastern regions of Uganda coupled with appropriate funding is urgently needed. Similarly, by comparing the traffic injury indicators between Uganda and other countries, especially the USA and Norway, this ought to avail Uganda with new insights on how these countries succeed in maintaining relatively low rates of RTIs despite being highly motorized countries.

**Limitations and Strengths**

a) The study only targeted free-of-cost articles which were accessible via the Tehran University of Medical Sciences Digital Library. For that reason, some insightful articles relevant to our study might have been missed. To overcome this, we manually hand-searched for other articles using the Google” search engine.

b) The scope of the majority of the trauma etiology related studies were city-based and hospital dependent. Their findings are likely to be narrow in elucidating detailed facts on RTIs in Uganda. In this regard, official traffic crashes and annual crime reports for example, from the UPF or WHO are more preferable to overcome this limitation.

c) By comparing Uganda’s injury patterns and road safety with the U.S, Norway, India, Ghana, Tanzania, Turkey and Iran in the two studies, we observed that the findings might have been misrepresented. This is because the injury and road safety indicators plaguing the six countries were of varying degrees compared to those affecting Uganda. Besides, all countries are at different levels of political and socio-cultural development.

d) The fact that, the articles that were published in 2011 and onwards were only eligible for inclusion in our study, to a certain degree their findings might have narrowed our scope of review and subsequent discussions. Therefore, similar studies may be needed to proceed this study which widen the inclusion criteria beyond the published articles.

e) It should be noted that, there are other respective factors responsible for the deaths of RTI victims other than ineffective emergency medical care for instance, the demographic characteristics of victims, road conditions and others. However, none of these aspects were reported in the findings of the articles that were reviewed. We again agitate for a widening the inclusion criteria for any other similar studies that will proceed this study.

Our study is the first systematic review exploring the trend of traffic crashes in Uganda. Its analysis focuses on different dynamics of road traffic trauma which include among the others: intervention, etiology, care, mortality and morbidity, registry and communication technologies, cost, and treatment. This is the paper's real strength despite the limitations mentioned above.

## Conclusion

Within the timeframe of five years (2011 - 2015) that represents the implementation of the United Nations Decade of Action for Road Safety, peripheral measures to reduce RTIs have been undertaken in Uganda. However, before being scaled-up, they essentially need to be premised on available research evidence gathered from all concerned stakeholders. So far, the various response actions aimed at reducing the burden of RTIs underscored in the reviews are to be lauded. Indeed, they are in tandem with the five pillars of the United Nations Decade of Action for Road Safety 2011–2020. However, in our view based on our analysis and agency, much more needs to be done to respond to the burden of RTIs. Now we have reached the fifth year of the Decade of Action for Road Safety and we only have remaining five years for its implementation, rigorous research beyond Kampala and/or the central region to other parts of Uganda is greatly needed. Much more needs to be done to promote the road safety education and awareness, fixing affordable trauma care centers and putting in place a national traffic crash database system. If we are to attain the five pillars enshrined in the United Nations Decade of Action for Road Safety, these opportunities to reduce RTIs greatly will surely need to be fully utilized.
